# Systemic Therapy for Merkel Cell Carcinoma: What’s on the Horizon?

**DOI:** 10.3390/cancers6021180

**Published:** 2014-05-16

**Authors:** Guilherme Rabinowits

**Affiliations:** Dana-Farber Cancer Institute, Harvard Medical School, Department of Medical Oncology, Center for Head and Neck Oncology, 450 Brookline Avenue, Boston, MA 02215, USA; E-Mail: guilherme_rabinowits@dfci.harvard.edu; Tel: +1-617-632-3090; Fax: +1-617-632-4448

**Keywords:** Merkel cell carcinoma, merkel cell polyomavirus, immunotherapy, targeted therapy

## Abstract

Merkel cell carcinoma is an aggressive neuroendocrine skin cancer that usually affects elderly patients. Despite being uncommon, incidence has been steadily increasing over the last two decades, likely due to increased awareness, better diagnostic methods and aging of the population. It is currently one of the most lethal cutaneous malignancies, with a five-year overall survival of approximately 50%. With the better understanding of the molecular pathways that lead to the development of Merkel cell carcinoma, there has been an increasing excitement and optimism surrounding novel targeted therapies, in particular to immunotherapy. Some of the concepts surrounding the novel targeted therapies and currently ongoing clinical trials are reviewed here.

## 1. Introduction

Merkel cell carcinoma (MCC) is an uncommon and aggressive neuroendocrine skin tumor thought to originate from the Merkel cells of the basal layer of the epidermis given its shared cytoplasmic dense-core neuroendocrine granules and keratin filaments (cytokeratin-20) expression characteristics [1,2]. It usually affects individuals in their 7th and 8th decades of life. Known risk factors include age older than 50 years, white skin, Merkel cell polyomavirus (MCV) infection, T-cell immune deficiency and ultraviolet exposure [3,4]. For those without distant metastasis at presentation, surgery with or without radiation therapy cure approximately half of them [5]. Unfortunately the other half will develop disease recurrence and die of metastatic disease 1 to 2 years later, with only 18% of patients alive at 5 years [5,6]. MCC remains an orphan disease with no standard of care treatment for those with advanced disease. Because of the histologic and biologic similarities to small cell lung cancer, another high-grade neuroendocrine tumor, the same chemotherapeutic agents are commonly used either alone or in combination. Although response rates reported to polychemotherapy are 60%–75%, median duration of response is only 8 months with a toxic death rate around 4%–7% for this patient population [7,8]. Newer, more effective and less toxic therapies are clearly needed. The recent discovery of a human polyomavirus (MCV) clonally integrated in MCC cells explains, at least partially, why patients with T cell deficiency have a higher risk of developing MCC when compared to the general population [4,9]. Rodig *et al.*, utilizing a novel mouse monoclonal antibody (Ab3) with increased sensitivity for a MCV large T antigen fragment identified MCV in 97% of their samples, in contrast to 81% found with the previously described murine monoclonal antibody CM2B4 [10]. This suggests that MCV in MCC is more common than previously reported. In fact, using improved polymerase chain reaction-based methods against both the large T and small T antigens, viral DNA was detected in 100% of the tumors tested, supporting the notion that MCV is a major contributor to the pathogenesis of MCC [10]. Despite the increased risk associated with immunosuppression, most of the newly diagnosed patients have no history of immune dysfunction, suggesting that tumor cells are capable of hiding and escape from the immune system [3]. Mechanisms of immune evasion include the tumor cell ability of “immunoediting”, down- and/or up-regulating molecules required for immune recognition and suppression, respectively [11]. This is supported by evidence that tumor cells from immunodeficient mice are more immunogenic than those from immunocompetent mice [11]. With a better understanding of the biology of this disease, significant interest has risen in testing targeted/immune therapies in MCC. Here we review the rationale for therapies currently ongoing testing in MCC or underway.

## 2. Cytotoxic Therapy

Given the rarity of this disease and limited number of prospective trials, most of the data is obtained from a single institution, retrospective studies of small number of patients and meta-analysis. Commonly used agents include platinum, etoposide, anthracycline, cyclophosphamide, vincristine, bleomycin and 5-fluorouracil either alone or in combination. No randomized studies have compared different chemotherapy regimens and most of the studies evaluating chemotherapy have a mixture of patients with metastatic and locally advanced disease, where chemotherapy was used in the adjuvant setting. Voog *et al.* performed a literature review on chemotherapy for patients with MCC [7]. It was a very heterogeneous group with 107 (75 with distant metastasis) patients undergoing 42 different chemotherapy regimens. Cyclophosphamide or ifosfamide-containing regimes were given to 60 patients (56%), anthracycline-containing regimens to 53 patients (49%), platinum-containing regimens to 27 patients (25%), 5-fluorouracil-containing regimens to 14 patients (13%) and other regimens to 13 patients (12%). For those with metastatic disease, the objective response rate and median overall survival was 57% and 9 months, respectively. Although response rates appeared to be better for cisplatin and adriamycin combination therapy, and 5-fluorouracil containing (versus non-containing) regimens, none was associated with significantly superior survival. In addition, 16% of the patients aged 65 and older died of treatment-related toxicity. Interestingly, two out of 14 patients with metastatic disease who achieved a complete response after first line chemotherapy were still alive without evidence of disease 45 and 120 months after the beginning of chemotherapy. Given the significant toxicity associated with cytotoxic therapy for these patients, increased efforts should be made to identify those who will likely derive the most benefit from cytotoxic therapy and spare those unlikely to benefit from the toxicity related to it.

## 3. B-Cell Lymphoma 2 (Bcl-2)

Bcl-2 oncoprotein blocks programmed cell death (or apoptosis) and contributes to tumor growth [12]. Moll *et al.* compared Bcl-2 protein expression between Merkel cells and MCC, and demonstrated a higher and more homogeneous protein expression in tumors, suggesting a role of this anti-apoptotic protein in the pathogenesis of this disease [13]. Oblimersen sodium (G3139; Genasense, Genta Incorporated, Berkeley Heights, NJ, USA), a phosphorothionate antisense oligonucleotide that blocks the production of Bcl-2 protein, was tested in severe combined immunodeficiency mouse xenotransplantation model for human MCC. Results were very encouraging with decreased Bcl-2 expression, increased apoptosis and reduction of tumor growth seen in those treated with oblimersen in comparison to control oligonucleotides or cisplatin [14]. This led to a multicenter phase 2 trial testing this agent in 12 patients with recurrent or metastatic MCC [15]. Results of this trial were disappointing revealing no objective responses and stable disease in only 3 patients. In an attempt to evaluate differences in Bcl-2 expression between MCV positive and negative MCC, and correlate its expression with prognosis, Sahi *et al.* analyzed 116 MCC specimens with clinical data. Eighty-five percent of the MCC were Bcl-2 positive [16]. No significant difference in MCV DNA occurred between the Bcl-2 positive and negative tumors. In contrast to what was previously thought, Bcl-2 negative tumors had more advanced stage at presentation and worse prognosis in contrast to Bcl-2 positive tumors. These results suggest that tumor progression may be due to mechanisms that are independent of Bcl-2. On another study, Lasithiotaki *et al.* evaluated the gene expression analysis of both MCV positive and negative non-small cell lung cancer samples [17]. Downregulation of Bcl-2 gene and increased expression of BRAF gene and its phosphorylation were seen in MCV positive samples in comparison to MCV negative samples, further supporting the notion that other genomic abnormalities may contribute to the pathogenesis of MCV-related disease.

## 4. KIT

KIT (CD117), also known as mast/stem cell growth factor (SCF) receptor, is a proto-oncogene. Co-expression of KIT and its ligand stem cell factor has been reported in MCC suggesting an autocrine, ligand-dependent activation of KIT [18,19]. Experiments with MCC-1 cell lines demonstrated phosphorylation and activation of extracellular receptor kinase (ERK)1/2 and AKT in parallel with KIT autophosphorylation by SCF, leading to cell survival and proliferation [20]. Imatinib mesylate (Gleevec^®^, STI-571, Novartis, Basel, Switzerland) is a small molecule tyrosine kinase inhibitor against bcr-abl, platelet-derived growth factor receptor (PDGFR) and KIT. The frequent expression of KIT in MCC led the Southwest Oncology Group to evaluate the activity of imatinib 400 mg orally daily in 23 MCC patients [21,22]. Only one patient had a partial response and three had stable disease. No complete responses were seen. The median progression-free survival and overall survival were one month (95% CI: 1–2 months) and 5 months (95% CI: 2–8 months), respectively. DNA sequencing performed in only two patients (one non-responding and one with stable disease) revealed no KIT mutation. Other studies have confirmed the lack of KIT activating mutations in MCC which may explain the poor responses seen to imatinib therapy [18,23]. There is one case reported in the literature of a complete response in a patient with MCV positive-MCC after imatinib therapy [24]. It is unclear why this patient responded but an activating mutation in either KIT or PDGFR may have been present to explain that.

## 5. Somatostatin Analogue

Octreotide (Sandostatin, Sandoz Pharmaceuticals, Basel, Switzerland) is a biologically fully active octapeptide with structure and activities similar to those of the native hormone somatostatin. Somatostatin and analogues have been shown to inhibit angiogenesis and to have anti-secretory and anti-proliferative effects in both functional and non-functional tumors [25,26]. Pyronet *et al.* suggested that these effects may be mediated by different somatostatin receptors/signaling pathways, and on the specific target cell intracellular signaling [27]. A direct correlation between somatostatin receptor type 2 (sst2) protein expression and tumor response to octreotide therapy has been shown [28]. Expression of sst2 has been demonstrated in MCC [29], and long-lasting complete responses in patients with MCC expressing somatostatin receptor receiving octreotide analogues have been reported [30,31]. A phase 1, dose escalation study evaluating another somatostatin analogue, pasireotide (SOM230, Novartis) safety, pharmacokinetics, and anti-tumor activity in patients with metastatic disease is currently ongoing (www.clinicaltrials.gov identifier number: NCT01652547) (Table 1). A peptide receptor radionuclide therapy with radiolabeled somatostatin analog (177Lu-DOTATATE) is also under investigation for somatostatin receptor-expressing neuroendocrine tumors (NCT01237457) (Table 1).

**Table 1:** Clinical trials currently recruiting MCC patients.

Clinicaltrials.gov identifier	Phase	Investigational Agent	Targets/Mechanism of Action
NCT02054884	2	F16-IL2 ± Paclitaxel	Tenascin-C *vs.* Microtubule disassembly inhibitor
NCT02036476	2	Cabozantinib	VEGFR2, MET
NCT01237457	2	177Lutetium-DOTA-Octreotide	Somatostatin Analogue
NCT01440816	2	Intratumoral IL-12	Immune stimulation
NCT02035657	1	GLA-SE	Toll-like Receptor 4 analogue
NCT01652547	1	Pasireotide	Somatostatin Analogue
NCT01758458	1	Adoptive immunotherapy	Autologus CD8 + Antigen-specific T cells
NCT01375842	1	MPDL3280	Anti-PD-L1

## 6. Survivin

Survivin (or baculoviral inhibitor of apoptosis repeat-containing-5), encoded by the BIRC5 gene, is a member of the inhibitor of apoptosis family. Its expression in tumors correlates with metastatic spread, tumor invasiveness and poor prognosis associated with chemotherapy resistance [32,33]. Indeed, survivin nuclear expression in MCC has been correlated with an aggressive clinical course [34]. Survivin expression is induced by MCV large T antigen and is critical to MCC survival. Arora *et al.* demonstrated a seven-fold increase in mRNAs for survivin in MCV-related *versus* non-related MCC [35]. Upon T antigen knockdown in several MCV-positive-MCC cell lines, both transcript and protein levels of survivin decreased, and knockdown of survivin resulted in cell death [36]. YM-155 (or Sepantronium bromide), binds to interleukin enhancer binding factor 3 (ILF3), ultimately decreasing E2F1/2-mediated transcriptional activation of survivin [36]. Dresang *et al.* tested YM-155 in MCV positive-MCC xenografts in non-obese diabetic, severe combined immunodeficient–gamma interleukin-2 receptor null mice [37]. The drug was shown to be cytostatic in three out of four xenografts and its efficacy was enhanced by extending the duration of treatment as well as by increasing the YM-155 dosage. In addition, the degree of efficacy was cell line dependent [37]. Other survivin inhibitors are currently undergoing testing in both pre-clinical and clinical settings. Clinical trials evaluating the activity of these agents alone or in combination with cytotoxic therapy in MCC are eagerly expected.

## 7. Antibody-Drug Conjugates (ADCs)

ADCs were developed in an attempt to optimize intra-tumoral drug delivery while sparing non-targeted tissues from the drug-related toxicity. It consists of an antibody, a cytotoxic drug and a linker that attaches the two. Once the ADC binds to the antigen expressed on the tumor cell surface, it undergoes internalization through a process called endocytosis [38]. Once internalized, the ADCs are commonly delivered to lysosomes where the antibody is degraded and the cytotoxic agent is released to bind to its pharmacological target [38]. Two ADCs are currently marketed for cancer treatment: Brentuximab vedotin (SGN-35, Adcetris^®^, Seattle Genetics and Millenium/Takeda, Cambridge, MA, USA), against cluster of differentiation (CD)30 for relapsed Hodgkin and anaplastic large cell lymphomas, and Trastuzumab emtansine (TDM-1, Kadcycla^®^, Genentech and Roche, South San Francisco, CA, USA) for HER-2 positive metastatic breast cancer that received prior taxane and trastuzumab therapies. Many other ADCs are under clinical testing. Virtually all MCC express CD56, which makes it an attractive target [39]. Lorvotuzumab mertansine (BB-10901, IMGN901, Immunogen, Waltham, MA, USA) is a conjugate of the cytotoxic maytansinoid, DM1, and the CD56-binding antibody, lorvotuzumab. A phase 1 dose escalating trial with this conjugate was performed in solid tumors. Once the maximal tolerated dose was established, an expansion cohort was performed to assess this ADC in patients with relapsed or refractory small-cell lung cancer, ovarian cancer and MCC [40]. On a preliminary report, two out of 14 MCC patients achieved a complete response. Updated efficacy and safety data are pending. Further studies evaluating this compound in MCC are anxiously awaited.

## 8. Phosphoinositide 3-kinase (PI3K)/Mammalian Target of Rapamycin (mTOR) Pathway

mTOR is a serine/threonine protein kinase of the PI3K-related kinase protein family. It regulates cell growth, proliferation, motility, survival, protein synthesis and transcription [41]. Lin *et al.* demonstrated mTOR pathway activation and impaired autophagy in MCC cell lines and tumor tissues. Moreover, mTOR inhibition decreased cell proliferation and induced autophagy and cell death in human MCC cells [42]. Nardi *et al.* detected PIK3CA (phosphatidylinositol-4,5-bisphosphate 3-kinase, catalytic subunit alpha) activating mutations in six out of 60 MCC tumors, and demonstrated sensitivity of MCC cell lines harboring PIK3CA mutations to PI3K pathway inhibitors [43]. These studies suggest that patients with PI3KCA mutant MCC may benefit from PI3K pathway inhibitors. Phase 1 clinical trials evaluating PI3K pathway inhibitors in refractory solid tumors are currently available and consideration should be taken to include MCC patients on these studies.

## 9. Angiogenesis

Vascular endothelial growth factor receptor (VEGFR) is a tyrosine kinase receptor involved in angiogenesis. VEGFR type 2 (VEGFR2) has been shown to be overexpressed in MCC and its overexpression has been correlated with worse outcome [44,45]. A single case has been reported in the literature demonstrating activity of Pazopanib (Votrient^®^, GlaxoSmithkline, Brentford, UK), an oral multikinase inhibitor against VEGFR, PDGFR and KIT, in a patient with metastatic MCC that progressed after cytotoxic therapy [46]. A 1432T > C mutation in PDGFR*-*α (ser478pro) gene was found in three tumor samples (this patient and two others with MCC), as well as in their germline DNA. A previous study has also identified that single heterozygous base change in exon 10 of the PDGFR gene in 3 of 9 MCCs, suggesting this may be an activating mutation and possibly a predictive biomarker for tyrosine kinase inhibitors [47]. Cabozantinib (Cometriq^®^, XL-184, Exelixis, South San Francisco, CA, USA) is an oral multikinase inhibitor preferentially against VEGFR2 and c-MET (mesenchymal-epithelial transition factor). It was recently approved for treatment of patients with progressive, metastatic, medullary thyroid cancer (another neuroendocrine tumor) after a phase 3 clinical trial demonstrated an improvement in the median progression-free survival compared to placebo (11.2 months *versus* 4 months, *p* < 0.001) [48]. MCC is known to overexpress paired box gene 5 (PAX-5), which controls c-MET [49]. Synergistic effects have been demonstrated with dual VEGFR2/c-MET inhibition [50]. A phase 2 trial evaluating the activity of cabozantinib in MCC is currently recruiting patients (NCT02036476) (Table 1).

## 10. Immunotherapies

### 10.1. Cytotoxic T-Lymphocyte Antigen 4 (CTLA-4, CD152)

CTLA-4 is a member of the CD28 family of receptors and ligands. The CD28 family contains a number of immunological checkpoints to attenuate the immune response caused by an inflammatory process (e.g, infection or neoplastic) and protects collateral tissue damage [51]. CTLA-4 is found on the surface of T cells, inhibiting its activation. Both CTLA-4 and CD28 share the same ligands: CD80 and CD86, which are present in antigen presenting cells and the T cells themselves. CTLA-4 counteracts the co-stimulatory activity of CD28 by competing for binding and its higher affinity for the shared ligands. Genetic ablation of CTLA-4 results in profound lymphoproliferation, particularly of the CD4^+^ T cells [52]. A phase 3 trial evaluating ipilimumab, a fully human monoclonal antibody against CTLA-4, in metastatic melanoma, resulted in prolonged overall survival compared to a peptide vaccine against the melanosomal gp100 antigen [53]. Based on these results, ipilimumab was approved for treatment of patients with metastatic melanoma. Importantly, 15% of the patients developed grade 3–4 toxicity, mostly immune-related, and death in seven patients [53]. A dense lymphocytic infiltrate of T-cells has been reported in cases of spontaneous regression of MCC after biopsy, strengthening the hypothesis of an immune-mediated process in the pathogenesis of MCC [54,55]. Moreover, Paulson *et al.* have demonstrated that intratumoral infiltration of CD8^+^ lymphocytes and lack of systemic immune suppression are independent predictors of improved survival among MCC patients [56,57]. Clinical trials testing anti-CTLA-4 in MCC are eagerly expected, and risks/benefits should be carefully weighted on this patient population given the drug-related toxicity previously reported. For unclear reasons, a phase 2 trial evaluating the activity of ipilimumab in patients with metastatic MCC was recently withdrawn prior to initiation of patient accrual (NCT01913691).

### 10.2. Programmed Death 1 (PD-1)

PD-1 is another T-cell co-inhibitory receptor of the CD28 family of receptors and ligands. It is expressed in T cells, B cells and NK cells, upon activation, and has 2 ligands: PD-L1 is expressed in both hematopoietic (B, T, myeloid and dendritic cells) and non-hematopoietic cells, and non-lymphoid organs; PD-L2 is expressed in antigen presenting cells (macrophages and dendritic cells) and more recently was found to be expressed in T cells [58,59]. Engagement of PD-1 with its ligands, inhibits T-cell receptor signaling and dowregulates the expression of anti-apoptotic molecules and pro-inflammatory cytokines [59]. Upregulation of PD-L1in peripheral tissues, in response to immune stimulation (interferon-γ), protect them against the collateral damage caused by the activated T-cells [60]. PD-1 is expressed by a large proportion of tumor-infiltrating lymphocytes (TIL) [61]. PD-L1 is expressed in many tumors and its expression correlates with poor prognosis [62]. Indeed, Taube *et al.* described increased expression of PD-L1 in melanoma cells immediately adjacent to TILs while tumors with minimal TIL infiltration were less likely to express PD-L1, suggesting that tumor cells protects themselves against immune-mediated destruction, at least partially, by inactivating effector T cells through PD-1/PD-L1 engagement [63]. Results of a phase 1 clinical trial evaluating anti-PD-1 in 296 patients with refractory solid tumors, revealed disease responses in melanoma (28%), renal cell carcinoma (27%) and in non-small cell lung cancer (18%) [64]. Thirty-six percent of the patients with PD-L1 expression responded in contrast to none without it. Grade 3-4 immune-related events occurred in 6% of the patients, with fatal pneumonitis seen in 3 cases. Afanasiev *et al.* demonstrated that circulating MCV-specific CD8 T cells and MCC-infiltrating lymphocytes express higher levels of PD-1 compared to T cells specific to other human viruses [65]. As seen in melanoma, higher PD-L1 expression was correlated with TIL infiltration, suggesting that anti-PD-1/PD-L1 inhibitors may be a therapeutic rationale for these patients [65]. In another study, Lipson *et al.* analyzed 49 tumors and demonstrated PD-L1 expression in 50% of the MCV positive- and none in the MCV negative-MCCs [64]. In addition, PD-L1 expression correlated with CD8^+^ TILs and it was shown to be a positive prognostic factor, again suggesting that enhancing the immune response with PD-1/PD-L1 blockade may prove to be a successful therapy for these patients [66]. A multicenter phase 1 clinical trial testing MPDL3280A (Genentech), a human monoclonal antibody against PD-L1, in refractory solid and hematologic malignancies, including MCC is currently recruiting patients (NCT01375842) (Table 1).

### 10.3. Adoptive Immunotherapy

Adoptive immunotherapy involves the process of isolating TILs or virus-specific lymphocytes, expansion of these cells in the lab and infusion of the autologous lymphocytes back to the patients. Rosenberg *et al.* described durable complete responses in 93 heavily pretreated patients with metastatic melanoma using T cell transfer immunotherapy [67]. Lymphocytes from resected metastatic melanoma lesions were grown in the lab with high-dose interleukin(IL)-2. Prior to receive TIL infusion, all patients received a non-myeloablative lymphodepleting regimen with or without total body irradiation (TBI) and autologous stem cell transplant. Overall response rate was 56%; 22% of the patients achieved a complete response (CR). Three- and five-year survival rates were 36% and 29%, respectively, with 93% of those in CR alive in 5 years. On another study, 10 patients with Epstein-Barr virus (EBV)-positive poorly or undifferentiated nasopharyngeal carcinoma (4 with high risk of relapse and 6 with relapsed/refractory disease) underwent adoptive transfer of cytotoxic T lymphocytes specific for the EBV antigens [68]. Two out of six patients with resistant or refractory disease had complete and sustained remissions, one had a partial response and another one stable disease. The four patients who were in remission at the time of the infusion, remained disease free after 19 to 27 months. Treatment was well tolerated. Lyngaa *et al.*, through a high-throughput flow cytometry-based platform developed for T-cell epitope identification, characterized MCV-specific CD8 T-cell epitopes in large T antigen, small T antigen and polyomavirus capsid protein 1 (VP1) [69]. Although T cells recognizing the MCV-encoded VP1 were present in both MCC patients and healthy donor, T-cell responses to the oncogenic proteins large and small T antigens were exclusively detected in MCC patients, representing ideal targets for immunotherapy. Indeed, large T antigen-specific CD8 T cells effectively killed MCV positive-MCC cell lines [69]. A phase 1/2 trial is currently investigating this approach in metastatic MCC (NCT01758458) (Table 1).

### 10.4. Intratumoral Therapy

Electrocorporation is the process that utilizes an electric charge to facilitate the entry of macromolecules (chemotherapy or immunotherapy) into the cell. Results of a phase 1 dose escalation trial evaluating IL12 plasmid electrocorporation in 24 patients with metastatic melanoma have been reported [70]. Treatment was well tolerated with no dose limited toxicities identified. Interestingly, two out of 19 patients developed complete regression of all metastasis, including those untreated, without any other systemic therapy administered, suggesting a systemic effect. Eight additional patients had stable disease or partial response. A phase 2 clinical trial testing this therapy in metastatic MCC is currently ongoing (NCT01440816) (Table 1).

### 10.5. F16-IL2

The immunocytokine F16-IL2 consists of the human monoclonal antibody F16 specific to the extradomain A1 of tenascin-C fused to human IL2 [71]. Tenascin-C is part of the stroma of most solid cancers, and plays a role in enhancing proliferation, invasion and angiogenesis during tumorigenesis and metastasis [72]. Marlind *et al.* tested its therapeutic performance with and without chemotherapy in a human breast cancer xenograft model [71]. When used as monotherapy, F16-IL2 had a superior therapeutic benefit compared with unconjugated recombinant IL2. When F16-IL2 was combined with low and high dose paclitaxel or high (but not low) dose doxorubicin, a synergistic effect was demonstrated with a dramatic increase of survival time observed when compared to single agent therapy [71]. Pedretti *et al.* conducted a similar study evaluating the therapeutic activity of F16-IL2 and temozolamide (alone or in combination) in murine models of subcutaneous and intracranial glioblastoma [73]. In the subcutaneous model, monotherapy with F16-IL2 led to minor tumor growth retardation. Despite a strong tumor regression seen on the temozolomide group, response duration was short. By contrast, mice treated with the combination therapy underwent complete remission 40 days after beginning of therapy, and remained disease free for over 160 days. In the intracranial model, the combination treatment was also more efficacious, resulting in 73% decrease in tumor volume 25 days after the start of treatment, as well as in longer survival of the animals. In both xenograft models, there was a selective accumulation of F16-IL2 around tumor vascular structures, and the recruitment of immune effector cells into the tumor lesions, but not in normal organs of the same mice [73]. A phase 2 randomized trial is currently testing F16-IL2 with or without paclitaxel in patients with metastatic MCC (NCT02054884) (Table 1).

## 11. Glucopyranosyl Lipid Adjuvant-Stable Emulsion (GLA-SE)

GLA-SE is a toll-like receptor (TLR)4 agonist. TLR agonists mimic pathogens, stimulating dendritic cells to produce T helper 1 cell-promoting cytokines, tumor necrosis factor-α (TNF-α), IL1β, IL6 and IL12 [74]. Maturation of dendritic cells by TNF-α is required for activation of both T helper cells and cytotoxic T lymphocytes [75]. IL12 increases interferon-γ production, augmenting T helper-1 cell response [76]. In an attempt to enhance the T helper 1 cell-mediated cytotoxic T lymphocytes response to influenza virus, Behzad *et al.* combined GLA-SE to split-virus vaccines to stimulate peripheral blood mononuclear cells of adult patients *in vitro*, and then challenged them with live influenza virus [77]. Results were very promising with activation of myeloid dendritic cells, producing high levels of T helper 1 cells-promoting cytokines, and increased interferon-γ:interleukin-10 ratio and the cytolytic response to influenza virus challenge. Based on these effects, a phase 1 feasibility study is currently testing GLA-SE in MCC patients (NCT02035657) (Table 1).

## 12. Conclusions

Significant progress has been made on the understanding of MCC pathogenesis since the identification of a clonal integration of a human polyomavirus in MCC approximately 5 years ago [3]. Different mechanisms of immune system evasion and new targeted therapies have been identified promoting significant excitement for those who manage this aggressive disease. To date, MCC remains an orphan disease. Cytotoxic agents are still considered first line therapy for those with metastatic disease despite the toxicity associated with it and the palliative intent of therapy. Patients should be encouraged to enroll in clinical trials with the hope that newer therapies will change the outcome of this devastating disease. Although immunotherapy trials are not an option for those immunosuppressed, the anti-angiogenesis, PI3K/mTOR inhibitors and somatostatin analogue trials are. Providers should not hesitate to refer patients for phase 1 trials, even as first line therapy, if there is a good rationale to test that drug for this disease. As noted above, preliminary results of a phase 1 trial with lorvotuzumab in refractory solid tumors revealed complete responses in 2 metastatic MCC patients. Finally, it is important to remember that MCC is a radiosensitive disease and radiation therapy should be considered for patients with symptomatic metastatic or recurrent disease.
